# Sex Partnership and Self-Efficacy Influence Depression in Chinese Transgender Women: A Cross-Sectional Study

**DOI:** 10.1371/journal.pone.0136975

**Published:** 2015-09-14

**Authors:** Xiaoshi Yang, Lie Wang, Chun Hao, Yuan Gu, Wei Song, Jian Wang, Margaret M. Chang, Qun Zhao

**Affiliations:** 1 Department of Social Medicine, School of Public Health, China Medical University, Shenyang, Liaoning Province, China; 2 Department of Medical Statistics and Epidemiology, School of Public Health, Sun Yat-sen University, Guangzhou, Guangdong Province, China; 3 Department of AIDS and STD Control, Shenyang Centers for Disease Control and Prevention, Shenyang, Liaoning Province, China; 4 Department of Clinical Laboratory, Liaoning Province Hospital of Chinese Armed Police Force, Shenyang, Liaoning Province, China; 5 Department of Clinical Medicine, China Medical University, Shenyang, Liaoning Province, China; 6 Takemi Program in International Health, Department of Global Health and Population, Harvard School of Public Health, Boston, Massachusetts, United States of America; UCSF, UNITED STATES

## Abstract

**Background:**

Transgender women often suffer from transition-related discrimination and loss of social support due to their gender transition, which may pose considerable psychological challenges and may lead to a high prevalence of depression in this population. Increased self-efficacy may combat the adverse effects of gender transition on depression. However, few available studies have investigated the protective effect of self-efficacy on depression among transgender women, and there is a scarcity of research describing the mental health of Chinese transgender women. This study aims to describe the prevalence of depression among Chinese transgender women and to explore the associated factors.

**Methods:**

A cross-sectional study was conducted in Shenyang, Liaoning Province of China by convenience sampling from January 2014 to July 2014. Two hundred and nine Chinese transgender women were interviewed face-to-face with questionnaires that covered topics including the Zung Self-Rating Depression Scale (SDS), demographic characteristics, transition status, sex partnership, perceived transgender-related discrimination, the Multidimensional Scale of Perceived Social Support (MSPSS) and the adapted General Self-efficacy Scale (GSES). A hierarchical multiple regression analysis was performed to explore the factors associated with SDS scores.

**Results:**

The prevalence of depression among transgender women was 45.35%. Transgender women with regular partners or casual partners exhibited higher SDS scores than those without regular partners or casual partners. Regression analyses showed that sex partnership explained most (16.6%) of the total variance in depression scores. Self-efficacy was negatively associated with depression.

**Conclusions:**

Chinese transgender women experienced high levels of depression. Depression was best predicted by whether transgender women had a regular partner or a casual partner rather than transgender-related discrimination and transition status. Moreover, self-efficacy had positive effects on attenuating depression due to gender transition. Therefore, interventions should focus on improving the sense of self-efficacy among these women to enable them to cope with depression and to determine risky sex partnership characteristics, especially for regular and casual partners.

## Introduction

Transgender women are socially excluded by the general community [1], which results in considerable psychological challenges. They usually suffer from mental health disorders such as psychological distress, and they may even resort to committing suicide [2–3]. Previous literature has reported that 80% of transgender women with gender dysphoria had a history of mental illness [4]. Depression is one of the most common disorders of psychological distress. The lifetime prevalence rate of depression in transgender women is disturbingly high, ranging from 48% to 62% [5–7]. A significant amount of research has revealed that transgender people experience higher levels of depression than the general population [8].

Several models have been used to understand the factors that can lead to depression in transgender women. The socio-ecological model shows that the variables that are proposed to predict depression in transgender women can be categorized into interpersonal factors (such as social support and sex partnership), intrapersonal factors (such as demographic characteristics, transition status and self-efficacy) and structural-level factors (such as social discrimination) [9]. According to the minority stress model [10], individuals who are of a sexual or gender minority may be rejected by family members, friends, and general society rather than being acknowledged, advocated, and supported. As a result, transgender individuals are more susceptible to receiving discrimination than are gay men, lesbians and bisexuals [11]. Transgender women in particular are more likely than transgender men to be evicted from their homes due to family discrimination [12]. The minority stress model also indicates that discrimination increases the rate of depression in the transgender population [10]. As a result, transgender women’s depression may be a result of discrimination, as reported in previous studies [13–14].

Transgender individuals are exposed to a variety of psychosocial challenges and have been found to be at an increased risk of loss of social support [8, 15]. Social support is defined as the provision of resources from others that are perceived to be helpful to the recipient and is the mechanism through which partners affect each other’s health and behavior [16]. Based on previous studies [8, 17, 18], social support seems to positively affect transgender individuals’ psychological well-being and the smoothness of their gender transition. However, social support for transgender individuals is quite scarce [17]. Transgender individuals with less social support might address psychological challenges more negatively, which may elevate their risk of developing depression [18–19].

Just as social support seems to significantly affect depression among transgender women, intrapersonal factors, such as self-efficacy, also appear to have an effect on the assessment of stressful situations, according to classical stress models elaborated by Lazarus and Folkman [20]. Studies on self-efficacy have yielded various findings with regard to how self-efficacy affects individuals’ distress. Self-efficacy is the positive capacity of an individual to manage stress and serves as a self-regulatory function by providing individuals with the capability to adapt their own thoughts and actions according to their environment [21]. Transgender individuals with higher self-efficacy are more likely to positively cope with the gender transition and have a lower likelihood of being depressed due to the adverse effects of gender transition.

Moreover, an individual’s transition status (such as the time spent living as a transgender women or the transition process) and sex partnership (such as having a regular partner or a casual partner or performing commercial sex work) as well as demographic characteristics may be related to depression. Previous studies have indicated that the longer transgender individuals were in their transition status, the more negative health outcomes they reported [17]. However, most recent studies have focused on transgender individuals’ hormone therapy, which is associated with their mental health [22]. These studies emphasize that sex partnership, including primary partners and outside partners, may influence sexual behavior choices for transgender individuals [16, 17] and may subsequently result in feelings of depression among these individuals. It is also well known that the high prevalence of sex work among transgender women is an important factor related to depression in these women [23]. A meta-analysis estimated that the rate of engagement in sex work among transgender women was 41.5% [14]. Another study revealed that nearly two-thirds (as many as 67%) of transgender women between the ages of 15 and 24 were engaged in sex work [23].

Consistent with previous research, we hypothesized that transition status, sex partnership, greater exposure to transgender-related discrimination, lack of social support and low levels of self-efficacy would be associated with depression. However, few studies have investigated the effects of protective factors, such as self-efficacy, against depression among transgender women based on the socio-ecological model, and there is a scarcity of research on the mental health of Chinese transgender women. The aim of this study was to assess levels of depression in this population and to explore how interpersonal factors (such as social support and sex partnership), intrapersonal factors (such as transition status and self-efficacy) and structural-level factors (such as social discrimination) influence depression among Chinese transgender women. This research will provide evidence regarding the identification of transgender women at high risk of emotional distress and will suggest effective management strategies to improve mental health among Chinese transgender women.

## Methods

### Study design and sample

A cross-sectional study was conducted in Shenyang from January 2014 to July 2014 using convenience sampling. This study was conducted in collaboration with the local non-governmental organization (NGO) that provides services for transgender women. Inclusion criteria included identifying as a transgender woman (any person who believed her male biological sex assigned at birth was in conflict with her gender identity), being at least 18 years old and living in Shenyang for at least three months. The faculty of the NGO provided the recruiting information in the community spaces and venues where transgender women congregated, including bars, nightclubs, beauty salons, street locations, health clinics, and community-based organizations as well as through their online website ‘Liaoning MSM web’. Thus, the participants in this study were recruited through community-based organizations, grassroots support groups, community outreach, and personal referrals by other transgender individuals. Individuals who expressed interest in the research were screened for eligibility, and those who met the inclusion criteria received appointments to meet a research assistant at the project office or at another convenient space that provided privacy. A total of 209 male-to-female transgender women fitting the above-mentioned criteria were enrolled in the study.

### Ethics Statement

The study protocol and informed consent form received ethics approval from the Committee on Human Experimentation of China Medical University. The transgender women who agreed to participate were asked to sign written informed consent forms and given face-to-face interviews by trained surveyors. Written informed consent concerning the conduct of the survey was obtained from each participant, with each participant using a nickname as a means of remaining anonymous. Each interview lasted approximately 20–30 minutes. Respondents were offered a gift card worth 50 Yuan (mobile phone rechargeable card) and a box of condoms to compensate them for their time and effort. The procedures followed were in accordance with and approved by the ethical standards of the Committee on Human Experimentation of China Medical University.

### Measurements

#### Assessment of depression

Depression was assessed with the Zung Self-Rating Depression Scale (SDS) [24], a standard assessment instrument using a four-point Likert scale that has been proven to accurately reflect subjective feelings of the severity of depression. The questionnaire, which has been validated and widely used in China, is composed of 20 questions with four possible responses: (1) never, (2) rarely/sometimes, (3) frequently and (4) always. Each item was scored from 1 to 4 according to severity. The raw score was standardized according to the following formula: standard score = int (1.25*raw score). The higher the SDS score, the more serious the depression disorder. A total standard score of 50 was set as a cut-off point to indicate depression. The internal consistency of the scale was good (Cronbach's alpha = 0.95).

#### Intrapersonal factors

*Demographic characteristics*: Demographic characteristics included age (<25, 25–29 or ≥30 years), gender (being entirely female or being androgynous), marital status (married or other, including unmarried/widowed/divorced/separated), educational level, and monthly income. Educational level was categorized as ‘≤junior middle school or below’, ‘senior middle school’ and ‘≥college’. Monthly income (RMB) was classified as ‘<3000 Yuan’, ‘3000–5000 Yuan’, and ‘≥5001 Yuan’. Gender was divided into two categories: ‘entirely female’ and ‘androgynous’. The category ‘entirely female’ refers to individuals with a completely female gender identity; the category ‘androgynous’ refers to individuals with both masculine and feminine characteristics who identify as being mentally between a woman and a man.

*Transition status*: Questions regarding transition status covered the following topics: the period of living as a transgender woman, feminine dress during daytime (yes/no), breast augmentation surgery (yes/no), and hormone use (yes/no). ‘Period of living as a transgender woman’ described the length of time an individual had existed as a transgender woman (the period from when new gender presentation or behavior commenced to the point of investigation). The period of living as a transgender woman was categorized into ‘≤2 years’, ‘3–4 years’, and ‘≥5 years’.

*Self-efficacy*: Self-efficacy was assessed by the adapted General Self-efficacy Scale (GSES) by Song Zhaoli [25]. The scale includes 10 items, and the responses range from strongly disagree (1) to strongly agree (4). The GSES was adapted from the English version [26], and higher scores indicated higher levels of self-efficacy. This scale provides an overall summary score and has been shown to have high reliability (Cronbach's alpha = 0.909) and good validity.

#### Interpersonal factors

*Sex partnership*: Sex partnership categories included having a regular partner or a casual partner, selling sex or buying sex. Transgender women were considered to have ‘regular partners’ if they had a regular partner in the past month. Individuals were considered to have ‘casual partners’ if they had casual partners other than commercial sex partners in the past month. ‘Selling sex’ was defined as being paid for commercially selling sex in the past month. ‘Buying sex’ was defined as buying commercial sex in the past six months.

*Perceived Social Support*: Perceived social support was measured by the Multidimensional Scale of Perceived Social Support (MSPSS) [27]. All the items were rated on a scale from 1 to 7. A total mean score was calculated, with higher scores indicating greater perceived support. This scale has been widely used in China [28]. The Cronbach's alpha in this study was 0.935, indicating good reliability.

#### Structural-level factors

*Perceived transgender-related discrimination from family*, *friends and society*: Perceived discrimination was assessed with three questions: *‘How serious is the discrimination from your family for being a transgender woman*?*’; ‘How serious is the discrimination from your friends for being a transgender woman*?*’;* and *‘How serious is the discrimination from society for being a transgender woman*?*’*. The responses for each item were (1) very serious/somewhat serious, (2) not sure, and (3) no discrimination at all.

*Statistical analysis*: The distributions of SDS scores among different categories of categorical variables were evaluated with t-tests and one-way analysis of variance (ANOVA) analyses. Correlations between the SDS scores and continuous variables were assessed using Pearson’s correlation. All the continuous variables were standardized to avoid multicollinearity [29] before performing the regression analysis. Hierarchical multiple regression (HMR) analysis was conducted to test the incremental variance by a set of independent variables. The SDS scores were used as dependent variables, and the independent variables were entered in the following steps: Step 1: demographic characteristics of the transgender women; Step 2: transition status; Step 3: sex partnership; Step 4: transgender-related discrimination; Step 5: social support; and Step 6: self-efficacy. The analysis proceeded in stages by successively including several blocks of independent variables in the regression model. The blocks of variables entered in later stages were thus tested for their extra contribution after the contributions of previously entered variables had been removed. The relative importance of the variables retained in the final multiple regression models contributed to the variance in the SDS scores, which was represented as the standardized β. Standardized parameter estimates were used to make comparisons of the magnitudes of the associations across independent variables. The fit of the model was assessed with the R^2^ value. Statistical analyses were performed with the Statistical Package for Social Science Version 11.5, and a two-tailed probability value of less than 0.05 was considered to indicate statistical significance.

## Results

### Description of the participants

The baseline characteristics of the participants are presented in Table 1. There were 209 sexually active transgender women in this study, of whom 131 (62. 68%) dressed in a feminine manner during the daytime, 37 (17.70%) used hormones, and 43 (20.57%) had a history of breast augmentation surgery. The mean age of the participants was 26.7±4.4 years old (ranging from 18 to 45 years old). The period of living as a transgender woman ranged from 1 year to 20 years, and the mean period of living as a transgender woman was 3.6±2.6 years. Overall, most transgender women (73.21%) had regular partners, and 55.50% of them had casual partners in the past month. More than half (56.94%) of the respondents were engaged in commercially selling sex in the past month. Approximately 17.70% of the interviewees bought sex in the past six months. The percentages of transgender women who experienced discrimination from family, friends and society were 70.33%, 31.10% and 75.12%, respectively.

**Table 1:** SDS scores in demographic characteristics and transition status of the transgender women.

Variables	N (%)	SDS
		Mean±SD
**Sex**		
Entirely female	97(46.41)	47.32±12.48
Androgynous	111(53.11)	47.31±13.18
Missing values	1(0.48)	
**Age**		
<25	72(34.45)	47.65±11.90
25–29	94(44.98)	46.96±13.06
≥30	43(20.57)	47.72±13.84
**Marital status**		
Married	193(92.34)	46.87±12.75
Others	16(7.66)	52.58±12.67
**Education level**		
≤ Junior middle school	50(23.92)	47.13±14.38
Senior middle school	103(49.28)	47.65±12.35
≥ College	54(25.84)	47.13±12.71
Missing values	2(0.96)	
**Monthly income**		
<3000	76(36.36)	48.81±12.02
3000–5000	81(38.76)	45.23±13.57
≥5001	52(24.88)	48.45±12.54
**Period of living as a transgender women**		
≤2 years	81(38.76)	45.33±12.93
3–4 years	74(35.41)	46.36±11.80
≥5 years	54(25.84)	50.96±13.19*
**Feminine dress in daytime**		
Yes	131(62.68)	47.23±12.90
No	78(37.32)	47.63±12.72
**Breast augmentation surgery**		
Yes	43(20.57)	49.00±12.57
No	166(79.43)	46.87±12.88
**Hormone use**		
Yes	37 (17.70)	51.40±11.93*
No	172(82.30)	46.41±10.86
**Regular partner**		
Yes	153(73.21)	49.43±12.43**
No	53(25.36)	41.48±12.40
Missing values	3(1.44)	
**Casual partner**		
Yes	116(55.50)	50.84±12.15**
No	81(38.76)	41.73±11.45
Missing values	12(5.74)	
**Selling sex**		
Yes	119(56.94)	47.92±12.59
No	90 (43.06)	46.47±13.20
**Buying sex**		
Yes	37(17.70)	49.34±13.35
No	145(69.38)	45.70±12.14
Missing values	27(12.92)	
**Family discrimination**		
Very serious/somewhat serious	147(70.33)	46.56±12.81
Not sure	43(20.57)	47.67±13.27
No discrimination at all	16(7.66)	51.61±11.12
Missing values	3(1.44)	
**Friend discrimination**		
Very serious/somewhat serious	65(31.10)	47.67±12.18*
Not sure	101(48.33)	49.13±12.11*
No discrimination at all	39(18.66)	41.84±14.14
Missing values	4(1.91)	
**Social discrimination**		
Very serious/somewhat serious	157(75.12)	46.94±12.54
Not sure	40(19.14)	47.30±13.90
No discrimination at all	9(4.31)	51.96±13.13
Missing values	3(1.44)	

Note

*P <0.05

** ***P* <0.01**(two-tailed).

### Description of the Depression

In this study, 45.35% of transgender women suffered from depression (SDS score higher than 50). The mean scores for depression based on the demographic characteristics are listed in Table 1. Transgender women who had a regular or casual partner reported significantly higher SDS scores (Mean±SD: 49.43±12.43, 50.84±12.15) than transgender women without a regular or casual partner (Mean±SD: 41.48±12.40, 41.73±11.45). Transgender women who used hormones reported significantly higher SDS scores (Mean±SD: 51.40±11.93) than transgender women who did not use hormones (Mean±SD: 46.41±10.86). Transgender women who reported never experiencing discrimination from friends had much lower levels of depression than transgender women who had experienced discrimination from friends. In addition, the level of depression increased with the period of living as a transgender woman. For example, individuals who had lived as transgender women for five years or longer had a higher level of depression than those who had lived as transgender women for less than five years. Differences in monthly income and in whether individuals dressed femininely during the daytime, had breast augmentation surgery, sold sex, bought sex, or experienced family discrimination and social discrimination were not statistically significant.

### Correlations between Depression and Related Factors

The results of the analyses for correlations between SDS scores and related factors are provided in Table 2. Social support and self-efficacy were negatively correlated with SDS scores (*P*<0.01). The period of living as a transgender woman was positively correlated with SDS scores (*P*<0.05).

**Table 2:** Correlation of SDS and related factors.

	M	SD	1	2	3	4	5
**Depression**	47.36	12.81	1				
**Age**	26.70	4.38	0.068	1			
**Period of living as a transgender women**	3.60	2.63	0.176*	0.334**	1		
**Social support**	59.31	14.97	-0.292**	-0.096	-0.134	1	
**Self-efficacy**	2.58	0.59	-0.195**	-0.016	-0.15	0.452*	1

Note

*P <0.05

** ***P* <0.01**(two-tailed)

### Predictors of depression

Table 3 shows the results of the hierarchical multiple regression models for SDS scores. Except for demographic characteristics, transition status and transgender-related discrimination (P values for all variables of blocks 1, 2 and 4 were not higher than 0.05), sex partnership, social support and self-efficacy contributed significantly to the variance in depression scores. A total of 33.9% of the variance was explained by the final regression model. The R^2^ changes indicated that the incremental variance explained by each block of variables was 2.5%, 4.3%, 16.6%, 3.3% 4.5%, and 2.7% for the demographic characteristics of transgender women, transition status, sex partnership, transgender-related discrimination, social support and self-efficacy, respectively. This study also indicated that the sex partnership of transgender women contributed most to the variance of SDS scores. In the final model of the HMR, the predictors of the extent of depression experienced by transgender women included whether the women had a regular partner or a casual partner and the extent of self-efficacy. In model 5, social support was also negatively associated with SDS scores.

**Table 3:** The hierarchical multiple regression models of SDS.

	SDS					
Variables	Model 1 (Beta)	Model 2 (Beta)	Model 3 (Beta)	Model 4 (Beta)	Model 5 (Beta)	Model6 (Beta)
**Block 1 Demographic characteristics**						
Age	0.061	0.008	0.055	0.061	0.035	0.040
Sex (entirely female vs. androgynous)	0.015	0.074	-0.034	-0.026	-0.088	-0.057
Marital status	0.365	0.229	0.210	0.234	0.286	0.359
Senior middle school vs. ≤junior middle school	-0.270	-0.265	-0.463	-0.368	-0.269	-0.188
≥College vs. ≤junior middle school	-0.137	-0.068	-0.139	-0.090	-0.130	-0.135
Monthly income	-0.082	-0.084	-0.035	-0.039	-0.049	-0.037
**Block 2 Transition status**						
Period of living as a transgender women		0.203	0.102	0.076	0.064	0.032
Feminine dress in daytime (yes vs. no)		0.186	0.198	0.109	0.161	0.091
Breast augmentation surgery (yes vs. no)		0.042	0.048	0.033	0.147	0.205
Hormone use (yes vs. no)		-0.388	-0.265	-0.185	-0.176	-0.250
**Block 3 Sex partnership **						
Regular partner in last month (yes vs. no)			-0.565**	-0.575**	-0.535**	-0.493*
Casual partner in last month (yes vs. no)			-0.571**	-0.548**	-0.481*	-0.559**
Selling sex in last month (yes vs. no)			-0.130	-0.093	-0.148	-0.120
Buying sex in last six months (yes vs. no)			-0.035	0.062	0.140	0.156
**Block 4 Transgender-related discrimination**						
Family discrimination				0.193	0.220	0.275
Friend discrimination				-0.231	-0.192	-0.176
Social discrimination				0.065	-0.087	-0.121
**Block 5 Social support**					-0.244**	-0.145
**Block 6 Self-efficacy**						-0.206*
**R** ^**2**^	0.025	0.068	0.234	0.267	0.312	0.339
**△R** ^**2**^	0.025	0.043	0.166	0.033	0.045	0.027

Note

*P <0.05

** ***P* <0.01**(two-tailed).

## Discussion

The results of this study revealed that a significant percentage of Chinese transgender women experienced high levels of depression (45.35%). This rate was much higher than the rates observed among Latina male-to-female transgender women who resided in Los Angeles, California (35.0%) [30], men who had sex with men in Foshan (34.8%) and Mianyang in China (37.8%) [31–32] and the Chinese general population in Xi’an (21.7%) [33]. Our result was consistent with previous studies that found that approximately half of transgender women reported depression during their lifetime [6, 8]. However, our result was significantly lower than that observed among transgender women in San Francisco (62.0%) [7]. Overall, depression appeared to be common among Chinese transgender women. Interventions focusing on depression among transgender women are therefore greatly warranted. In this study, the extent of the depression experienced did not rely solely on intrapersonal factors, such as the level of self-efficacy as perceived by individuals, but also on interpersonal factors such as sex partnership.

Among the independent variables examined in this study, sex partnership was comparatively important, which was indicated by a relatively high standardized β. Sex partnership played the most important part in explaining levels of depression, accounting for 16.6% of the observed variability in depression. Specifically, having a regular partner or a casual partner had robust associations with depression, whereas selling sex and buying sex were not associated with depression. With regard to regular partners, transgender women with regular partners in the past month suffered from higher levels of depression than those without regular partners did. The possible reasons may be that transgender women with regular partners may be anxious due to fear of losing their partners and, as a result, may have concealed their gender identity or presentation. Moreover, these transgender women may have experienced anxiety due to their partners’ discrimination against them for being transgender. Furthermore, they were more likely to engage in unprotected anal intercourse (UAI) and were less likely to abstain due to the expectation of a concordant HIV status between partners and loyalty by both partners. This ideology was consistent with a previous study of MSM with regular partners [34].

Having a casual partner also seemed to be significantly associated with depression, which was in accordance with a previous study [35]. Transgender women who had a casual partner in the past month were more likely to engage in high-risk sexual behaviors due to a lack of HIV status disclosure, limited HIV prevention and low social support. These factors resulted in an elevated risk of HIV among these individuals because sexual behavior with a casual partner could lead to the potential for HIV infection, resulting in extreme concerns about physical health and contributing to high levels of depression among these transgender women. In this study, approximately 79.43% of the transgender women were below the age of 30, were sexually active and were not often limited by social constraints and responsibilities [36]. Most of the participants engaged in risky behaviors, which contributed to concerns about HIV risk and an elevated risk of depression. Prevention efforts should target sex partnerships, especially for transgender women with regular or casual partners, and improve the understanding of relationship contexts and the way a regular or casual partner may influence depression.

An additional finding from this study was that self-efficacy was an important predictor of depression among transgender women, which supported previous studies that found that positive cognitive beliefs influenced the appraisal of stressful situations and psychological adjustment to challenges [37]. Self-efficacy is considered a crucial psychological construct that may influence a transgender individual’s confidence during transition and when attempting to overcome the barriers to living with a new gender identity. Self-efficacy may also influence emotional reactions to stressful situations, in accordance with previous studies [38–41]. Transgender women with a high sense of self-efficacy could control their negative emotions and strive for the positive aspects of transition when resources were threatened [22]. Additionally, they were able to compensate for transition-related stress and effectively take advantage of social support and resources to confront challenges and harassments. This ability allowed them to maintain positive emotions and address their distress.

Surprisingly, in the univariate analysis in this study, it was found that individuals who had lived as transgender women for a period equal to or more than five years had higher levels of perceived depression than those who had lived with the new identity for a shorter period of time. This result conflicts with the observations in Budge’s study [16]. A possible reason was that a longer period of living as a transgender woman corresponded to a higher frequency of harassment encountered and a lower amount of available social support, resulting in feelings of depression. Additionally, transgender women who used cross-sex hormones exhibited higher levels of depression than those who did not use hormones, which also conflicted with the results of a previous study [42]. The reason for this might be that most Chinese transgender women confront barriers to accessing gender-related health care; consequently, most of them use hormones without instruction from doctors, which contributes to their depression.

The present study has the limitation that it is cross-sectional research. Therefore, one cannot derive conclusions about the causality of the associations observed between transition and depression. Additionally, the transgender women in this study were selected by convenience sampling, which might limit the generalizability of this study to other populations. However, despite the limitations, this study has notable strong points. First, research on Chinese transgender women has been scarce. Second, this study had a high response rate, most likely because face-to-face interviews facilitated the collection of information.

## Conclusion

Chinese transgender women experience high levels of depression. The depression is best predicted by whether transgender women have a regular partner or a casual partner rather than by the women’s transition status, demographic characteristics or the transgender-related discrimination they experience. Moreover, self-efficacy has positive effects on attenuating depression resulting from a gender transition. Interventions to reduce transgender women's depression would be likely to benefit from prioritizing prevention efforts to improve self-efficacy to cope with depression and from determining risky sex partnership network characteristics, especially for transgender women with regular or casual partners.

## Supporting Information

S1 Dataset (XLS)

## List of Abbreviations

(SDS): The Zung Self-Rating Depression Scale
(MSM): men who have sex with men
(MSPSS): the Multidimensional Scale of Perceived Social Support
(GSES): the adapted General Self-efficacy Scale
(ANOVA): One-way Analysis of Variance
(HMR): Hierarchical Multiple Regression
(UAI): Unprotected Anal Intercourse

## References

[1] Abdullah MA, Basharat Z, Kamal B, Sattar NY, Hassan ZF, Jan AD, et al (2012). Is social exclusion pushing the Pakistani Hijras (Transgenders) towards commercial sex work? a qualitative study. BMC Int Health Hum Rights. 12: 32 12:32. 10.1186/1472-698X-12-32 23163979 PMC3534382

[2] Nuttbrock L., Rosenblum A., & Blumenstein R (2002). Transgender identity affirmation and mental health. International Journal of Transgenderism, 4 Available: http://www.wpath.org/journal/ www.iiav.nl/ezines/web/IJT/97-03/numbers/symposion/ijtvo06no04_03.htm.

[3] Yadegarfard M, Ho R, Bahramabadian F (2013) Influences on loneliness, depression, sexual-risk behaviour and suicidal ideation among Thai transgender youth. Cult Health Sex. 15(6):726–37. 10.1080/13691058.2013.784362 23659441

[4] Hepp U, Kraemer B, Schnyder U, Miller N, Delsignore A (2005) Psychiatric comorbidity in gender identity disorder. J Psychosom Res 58(3):259–261. 15865950 10.1016/j.jpsychores.2004.08.010

[5] Nuttbrock L, Hwahng S, Bockting W, Rosenblum A, Mason M, Macri M, et al (2010) Psychiatric impact of gender-related abuse across the life course of male-to-female transgender persons. J Sex Res 47(1):12–23. 10.1080/00224490903062258 19568976

[6] Nemoto T, Bödeker B, Iwamoto M (2011) Social support, exposure to violence and transphobia, and correlates of depression among male-to-female transgender women with a history of sex work. Am J Public Health 101(10):1980–1988. 10.2105/AJPH.2010.197285 21493940 PMC3222349

[7] Clements-Nolle K, Marx R, Guzman R, Katz M (2001) HIV prevalence, risk behaviors, health care use, and mental health status of transgender persons: implications for public health intervention. Am J Public Health 91(6):915–921. 11392934 10.2105/ajph.91.6.915 PMC1446468

[8] Budge SL, Adelson JL, Howard KA (2013) Anxiety and depression in transgender individuals: the roles of transition status, loss, social support, and coping. J Consult Clin Psychol 81(3):545–557. 10.1037/a0031774 23398495

[9] Reisner SL, Mimiaga MJ, Bland S, Mayer KH, Perkovich B, Safren SA (2009) HIV risk and social networks among male-to-female transgender sex workers in Boston, Massachusetts . J Assoc Nurses AIDS Care (5):373–86. 10.1016/j.jana.2009.06.003 19732696 PMC3677528

[10] Otis MD, Rostosky SS, Riggle E, Hamrin R (2006) Stress and relationship quality in same-sex couples. Journal of Social and Personal Relationships 23:81–99.

[11] Barrientos J, Silva J, Catalan S, Gomez F, Longueira J (2010) Discrimination and victimization: parade for lesbian, gay, bisexual, and transgender (LGBT) pride, in Chile . J Homosex 57(6):760–775. 10.1080/00918369.2010.485880 20582801

[12] Fletcher JB, Kisler KA, Reback CJ (2014) Housing status and HIV risk behaviors among transgender women in Los Angeles. Arch Sex Behav 43(8):1651–1661. 10.1007/s10508-014-0368-1 25190499 PMC4214608

[13] Clements-Nolle K, Marx R, Katz M (2006) Attempted suicide among transgender persons: the influence of gender-based discrimination and victimization. J Homosex 51(3):53–69. 17135115 10.1300/J082v51n03_04

[14] Herbst JH, Jacobs ED, Finlayson TJ, McKleroy VS, Neumann MS, Crepaz N (2008) Estimating HIV prevalence and risk behaviors of transgender persons in the United States: a systematic review. AIDS Behav 12(1):1–17. 17694429 10.1007/s10461-007-9299-3

[15] Darbes LA, Chakravarty D, Beougher SC, Neilands TB, Hoff CC (2012) Partner-provided social support influences choice of risk reduction strategies in gay male couples. AIDS Behav 16(1):159–167. 10.1007/s10461-010-9868-8 21221756 PMC3254781

[16] Cohen S (2004) Social relationships and health. Am Psychol 59(8):676–686. 15554821 10.1037/0003-066X.59.8.676

[17] Budge SL, Katz-Wise SL, Tebbe EN, Howard KAS, Schneider CL, Rodriguez A (2012) Transgender emotional and coping processes: Facilitative and avoidant coping throughout gender transitioning. The Counseling Psychologist. Advance online publication. 10.1177/0011000011432753

[18] Poteat T, Wirtz AL, Radix A, Borquez A, Silva-Santisteban A, Deutsch MB, Khan SI, etal (2015) HIV risk and preventive interventions in transgender women sex workers. Lancet. 17;385(9964):274–86. 10.1016/S0140-6736(14)60833-3 25059941 PMC4320978

[19] Pinto RM, Melendez RM, Spector AY (2008) Male-to-Female Transgender Individuals Building Social Support and Capital From Within a Gender-Focused Network. J Gay Lesbian Soc Serv 20(3):203–220. 20418965 10.1080/10538720802235179 PMC2858872

[20] Lazarus RS, Folkman S (1984) Stress, Appraisal, and Coping Springer: New York 163–175.

[21] Kreitler S, Peleg D, Ehrenfeld M (2007) Stress, self-efficacy and quality of life in cancer patients. Psychooncology 16(4):329–341. 16888704 10.1002/pon.1063

[22] Gooren LJ, Sungkaew T, Giltay EJ, Guadamuz TE (2015) Cross-sex hormone use, functional health and mental well-being among transgender men (Toms) and Transgender Women (Kathoeys) in Thailand. Cult Health Sex. 17(1):92–103. 10.1080/13691058.2014.950982 25270637 PMC4227918

[23] Hoffman B (2014) An Overview of Depression among Transgender Women. Depress Res Treat 2014:394283 10.1155/2014/394283 24744918 PMC3972927

[24] Zung WW (1965) A Self-Rating Depression Scale. Archives of general psychiatry 12: 63–70. 14221692 10.1001/archpsyc.1965.01720310065008

[25] Zewen Liu, Zaoli Song, Huashan Liu (2006) Psychological Researches on Job Search Behaviors. Advances in Psychological Science 14(4):631–635. [Article in Chinese].

[26] Barlow JH, Williams B, Wright C (1996) The Generalized Self-Efficacy Scale in people with arthritis. Arthritis Care Res 9(3):189–196. 8971228 10.1002/1529-0131(199606)9:3<189::aid-anr1790090307>3.0.co;2-#

[27] Zimet GD, Dahlem NW, Zimet SG, Farley GK (1988) The multidimensional scale of perceived social support. J Pers Assess 52(1):30–41. 10.1080/00223891.1990.9674095 2280326

[28] Jiang QJ (2001) Perceived social support scale. Chinese Journal of Behavioral Medical Science (10): 41–42. (In Chinese)

[29] Preacher KJ, Hayes AF (2008) Asymptotic and resampling strategies for assessing and comparing indirect effects in multiple mediator models. Behav Res Methods 40(3):879–891. 18697684 10.3758/brm.40.3.879

[30] Bazargan M, Galvan F (2012) Perceived discrimination and depression among low-income Latina male-to-female transgender women. BMC Public Health 12:663 10.1186/1471-2458-12-663 22894701 PMC3497862

[31] Jun Liu, Yanhui Cao, Zimian Liang, Yuan Li, Yi Yang (2012) Depressive symptoms and associated sexual behaviors among men who have sex with men in Foshan, Guangdong province. Chin J Epidemiol 33(5):483–487. [Article in Chinese]. 22883174

[32] Yi Wang, Hongbo Zhang, Xu jie Zhang Guanggui, Hongwu Yang, Jing Fan (2010) Survey of Depression Status Among Men Having Sex With Men. J Prev Med Inf 26,(8):597–600. [Article in Chinese].

[33] Kefeng Guo, Shan Guo, Juxiang Guan, WenQing Yang, Yinxing Zhu, Qunqiang Wu, et al (2005) Incidence rate of depressive symptoms and the prevalence rate of depression in the crowds living in the fringe area of Xi’an city. Chin J Clin Rehabil 9(16):4–5. [Article in Chinese].

[34] Wong CF, Schrager SM, Chou CP, Weiss G, Kipke MD (2013) Changes in Developmental Contexts as Predictors of Transitions in HIV-Risk Behaviors Among Young Men Who Have Sex with Men (YMSM). Am J Community Psychol 51(3–4):439–50. 10.1007/s10464-012-9562-2 23254866

[35] Sandfort T, Yi H, Knox J, Reddy V (2013) Sexual partnership types as determinant of HIV risk in South African MSM: an event-level cluster analysis. AIDS Behav 17 Suppl 1:S23–32. 10.1007/s10461-012-0294-y 22956229 PMC3529978

[36] Arnett JJ (2005) The developmental context of substance use in emerging adulthood. Journal of Drug Issues 35, 235–253.

[37] Nouwen A, Urquhart Law G, Hussain S, McGovern S, Napier H (2009) Comparison of the role of self-efficacy and illness representations in relation to dietary self-care and diabetes distress in adolescents with type 1 diabetes. Psychol Health 24(9):1071–1084. 10.1080/08870440802254597 20205046

[38] Jefferson K, Neilands TB, Sevelius J (2013) Transgender women of color: discrimination and depression symptoms. Ethn Inequal Health Soc Care 6(4):121–136. 25346778 10.1108/EIHSC-08-2013-0013 PMC4205968

[39] Korpershoek C, van der Bijl J, Hafsteinsdóttir TB (2011) Self-efficacy and its influence on recovery of patients with stroke: a systematic review. J Adv Nurs 67(9):1876–1894. 10.1111/j.1365-2648.2011.05659.x 21645040

[40] Curtis R, Groarke A, Sullivan F (2014) Stress and self-efficacy predict psychological adjustment at diagnosis of prostate cancer Sci Rep. 4:5569 10.1038/srep05569 24993798 PMC4081888

[41] Bandura A (1993) Perceived self-efficacy in cognitive development and functioning. Educational Psychologist, 28,117–148.

[42] Gooren LJ, Sungkaew T, Giltay EJ (2013) Exploration of functional health, mental well-being and cross-sex hormone use in a sample of Thai male-to-female transgendered persons (kathoeys). Asian J Androl. 15(2):280–5. 10.1038/aja.2012.139 23353716 PMC3739151

